# Peripheral nerve stimulation for essential tremor: a GRADE-assessed systematic review and meta-analysis

**DOI:** 10.1007/s10143-026-04394-8

**Published:** 2026-07-21

**Authors:** Mark Messak, Yusof Mohamed Omar, Nada Mosad, Omar Ahmed, Hamdy A. Makhlouf

**Affiliations:** 1 Faculty of Medicine, Helwan university, Helwan, Egypt; 2 Faculty of Medicine, Mansoura university, Mansoura, Egypt; 3 Faculty of Medicine, Al-Azhar University, Damietta, Egypt; 4 Faculty of Medicine, Minia University, Minia, Egypt; 5 Medical Research Group of Egypt, Negida Academy, Arlington, MA USA

**Keywords:** Essential tremor, Neuromodulation, Non-invasive therapy, Peripheral nerve stimulation, Systematic review, Meta-analysis

## Abstract

Essential tremor (ET) is a common movement disorder; its management remains challenging despite the availability of multiple treatment options. Pharmacological therapies are often limited in efficacy and tolerability, while surgical options, although effective, are invasive and expensive. Peripheral nerve stimulation (PNS) has emerged as a non-invasive neuromodulation approach. However, different stimulation protocols, small samples, and inconsistent outcome reporting preclude firm conclusions. Therefore, we performed a systematic review and meta-analysis to evaluate the efficacy and safety of PNS for ET. A literature search was conducted through PubMed, Scopus, Web of Science, and Cochrane CENTRAL databases till January 2026. The primary outcome was the change in tremor intensity, as measured by the TETRAS Performance Scale (TETRAS-PS). Secondary outcomes included functional disability, assessed by the Bain and Findley Activities of Daily Living (BF-ADL) scale, clinical improvement assessed using the clinical global impression – improvement (CGI-I) scale, as well as safety outcomes. Quality assessment was performed using the Risk of Bias-2 (RoB-2) and the NIH single-arm tools. Certainty of evidence was assessed using GRADE approach. A meta-analysis was performed, calculating the mean difference (MD) with 95% confidence intervals (CIs) using Review Manager 5.4.1. Twelve studies, involving 914 participants, met the inclusion criteria, including four RCTs. Our analysis of RCTs demonstrated that active PNS produced a statistically significant reduction in tremor intensity (TETRAS-PS; MD=-0.92, 95%CI [-1.63 to -0.21], P = 0.01, I2 = 0) and an improvement in functional outcomes (BF-ADL; MD=-1.90, 95%CI [-2.97 to -0.83], p = 0.0005, I2 = 0). Benefits were observed immediately and persisted for up to three months in longitudinal studies. Adverse events were generally mild and limited mainly to transient skin irritation; no device-related serious adverse events were reported. PNS was associated with short-term reduction in tremor severity and functional impairment in patients with ET, with an overall acceptable safety profile, suggesting that it may serve as a middle ground option between pharmacotherapy and invasive neurosurgical approaches. However, further larger and longer randomized trials with refined sham controls are needed to confirm its efficacy, durability, and optimize stimulation strategies. CRD420261282826.

**Supplementary Information:**

The online version contains supplementary material available at 10.1007/s10143-026-04394-8.

## Introduction

Essential tremor (ET) is one of the most common movement disorders, affecting about 0.9% of people worldwide [1]. It is characterized by kinetic and postural tremors in the upper limbs and, less often, affects the head, face, voice, trunk, and lower limbs, leading to significant interference with daily activities and a decreased quality of life [2]. Although the etiology of ET remains unclear, evidence from histological, imaging, electrophysiological, and computational studies suggests abnormal activity within the cerebello-thalamo-cortical (CTC) network. This circuit links the deep cerebellar nuclei to the motor cortex through the motor and sensory thalamus, particularly the ventral intermediate (VIM) nucleus [3, 4]. Abnormalities within this circuit likely underlie the impaired control of goal-directed movements observed in ET [5]. ET further contributes to a substantial economic burden, with affected patients incurring higher annual healthcare costs than controls ($17,560 vs. $13,237), driven by increased burden of comorbidities (mean 5.3 (3.2) VS 4 (3.3)) and higher prevalence of psychiatric disorders (45.9% vs. 31.1%) [6].

Pharmacological treatment with nonselective β-blockers (such as propranolol) and anticonvulsants (such as primidone) remains the first-line of treatment [7, 8]. However, their intolerable side effects, with reported discontinuation rates varied from 10 to 70% [9], and 30–50% non-response rates prompted consideration of invasive interventions, such as VIM deep brain stimulation (DBS), or magnetic resonance-guided focused ultrasound (MRgFUS) VIM thalamotomy [10, 11]. Although these procedures can achieve substantial tremor reduction up to 80% [12], patients are usually hesitant due to the invasive nature and the potential adverse events that could be associated, including infection, bleeding, and brain tissue damage [13]. Additionally, these procedures are costly and not suitable for all patients [14], highlighting the need for less invasive alternatives.

Early neurophysiological studies demonstrated that muscle afferent input can modulate neuronal activity within the VIM, providing a mechanistic basis for the modulation of central tremor networks by peripheral nerve stimulation (PNS) [15]. Building on this physiological foundation, non-invasive neuromodulation was reported by 2018, showing clinical improvement while avoiding the risks of surgical procedures and producing minimal systemic side effects compared with pharmacological therapies [16]. Subsequently, transcutaneous electrical nerve stimulation (TENS) was applied using wearable wrist-based transcutaneous devices that deliver patterned electrical stimulation to the median, ulnar, and radial nerves, synchronized to the patient’s tremor frequency [17]. Devices employing various stimulation paradigms and treatment durations were evaluated in larger, multicenter studies, supporting their broader clinical use in ET [18, 19]. More recently, artificial intelligence (AI)-driven algorithms have been integrated into PNS systems to optimize stimulation parameters in real time, adapting to individual tremor characteristics [20].

The initial single-arm studies showed that PNS is safe and effective for reducing hand tremors in many ET patients [21]. Later, randomized controlled trials (RCTs) comparing PNS with sham treatments produced mixed results. Pahwa et al. reported significant improvements in upper limb tremor severity, with a reduction in Tremor Research Group Essential Tremor Rating Assessment Scale (TETRAS) scores (*p* = 0.017), along with greater functional gains in activity of daily living (ADL) (49% vs. 27%) and higher Clinical Global Impression–Improvement (CGI-I) response rates (88% vs. 62%) in the stimulation group compared to sham treatment [22]. In contrast, Samiee et al. found no statistically significant differences over time between the intervention and sham groups in changes in TETRAS, CGI-I, or Bain & Findley Activity of daily living (BF-ADL) scores [23]. Due to these conflicting findings, it is important to clarify the overall impact of PNS on ET outcomes.

To the best of our knowledge, no previous systematic reviews have addressed this topic. Therefore, our systematic review and meta-analysis aim to evaluate the effectiveness of PNS by incorporating all available evidence about its efficacy and safety, thereby providing a more precise and reliable estimate of the therapeutic effect of PNS on ET.

### Methods

We followed the Preferred Reporting Items for Systematic Reviews and Meta-Analyses (PRISMA) guidelines while reporting this manuscript [24]. We were adherent to the Cochrane Handbook of Systematic Reviews of Interventions version 6.5 while conducting this study [25]. This study was prospectively registered on PROSPERO with the registration number CRD420261282826.

### Eligibility criteria

Studies were selected based on the PICOS framework (population, intervention, comparison, outcomes, and study design) [26]. Eligible studies included those targeting patients with ET, where PNS was used to treat upper extremity tremors by stimulating the median, radial, or ulnar nerves. Studies involving direct muscle stimulation were excluded. Eligible interventions included any form of peripheral electrical stimulation, including conventional PNS, AI-assisted transcutaneous peripheral nerve stimulation (AI-TPNS), Transcutaneous afferent patterned stimulation (TAPS), and intramuscular stimulation of muscle afferents.

The study was limited to clinical trials (CTs) and observational studies excluding post-market studies to maintain methodological consistency and focus on studies evaluating the intervention’s efficacy and safety. A minimum of one clinically relevant outcome related to motor symptoms, quality of life, or adverse effects, assessed using validated measurement tools, was required for inclusion, with no restrictions on follow-up duration or time points. We excluded studies not written in English, studies evaluating functional electrical stimulation devices, surgical approaches, animal model studies and preclinical studies, case reports and case series, cross-sectional studies, conference abstracts, protocols, commentary articles, post-analysis articles, and papers that included patients other than those with ET, unless their data were reported separately.

### Literature search strategy

We conducted three different searches of electronic databases, including PubMed, Scopus, Web of Science (WOS) and Cochrane CENTRAL. We focused on all publications that met our eligibility criteria until January 2026. For a precise search strategy, we used Medical Subject Headings (MeSH) terms and keywords related to “Peripheral nerve stimulation” and “Essential tremors” The full search strategy is described in Supplementary Table 1. To identify any relevant research missed by the database search, the reference lists of retrieved papers were also analyzed.

### Selection process & data extraction

Eligibility screening was performed in two steps using Rayyan software [27]. Before the first step, duplicates were identified and manually removed by Rayyan’s duplicate detection feature. The first step was the title and abstract screening of all retrieved papers. The second step was full-text screening for those who passed the first step of screening to assess their eligibility for meta-analysis. Two authors were responsible for this selection process. Any disagreements between the two authors were resolved by consensus.

The data were extracted independently using an online extraction form. Summary of the included studies comprised: study ID, location, design, sample size, treatment duration, follow-up points, device name, and nerve stimulated. Baseline data of the participants comprised age, sex and baseline of both TETRAS – Performance Scale (TETRAS PS) and BF-ADL scores. For both the TETRAS-PS and BF-ADL scores, the mean change from baseline was extracted for the meta-analysis model.

### Risk of Bias and certainty of evidence assessment

Two authors evaluated the quality of the included studies independently. We used the Risk of Bias-2 (RoB-2) tool for assessing the RCTs [28]. The RoB-2 tool comprises five bias domains: (1) Bias arising from the randomization process, (2) Bias due to deviations from intended interventions, (3) Bias due to missing outcome data, (4) Bias in measurement of the outcome, and (5) Bias in selection of the reported results. For visualizing our risk of bias assessment of RCTs, we used robvis web app [29].

For single-arm longitudinal studies, we used the NIH single-arm quality assessment tool [30]. The NIH single arm quality assessment tool for single arm studies comprises 12 questions assessing: (1) Study question, (2) Eligibility criteria and study population, (3) Study participants representative of clinical populations of interest, (4) All eligible participants enrolled, (5) Sample size, (6) Intervention clearly described, (7) Outcome measures clearly described, valid, and reliable, (8) Blinding of outcome assessors, (9) Follow up rate, (10) Statistical analysis, 11) Multiple outcome measures, and 12) Group-level interventions and individual-level outcome efforts. Based on NIH single arm scoring, Studies were classified as poor (< 50%), fair (50% – 75%), or low risk (≥ 75%).

The certainty of evidence for each outcome was assessed using GRADE framework, where evidence was rated as high, moderate, low, or very low certainty according to five domains includes: risk of bias, inconsistency, indirectness, imprecision, and publication bias [31, 32].

### Measures of treatment effect & heterogeneity assessment

Our primary outcome was evaluating the tremor severity, measured using TETRAS-PS, a clinical assessment of both tremor amplitude and motor performance [33]. Secondary outcomes included functional disability, assessed using the BF-ADL scale, a patient-reported measure of tremor-related disability in daily activities, and overall clinical improvement assessed using the CGI-I scale.

When the standard deviation (SD) of change in one of the outcomes was not provided, we calculated it from a 95% CI or by imputing SD for changes from baseline using a correlation coefficient (r) = 0.5 suggested by Cochrane Handbook for Systematic Reviews of Interventions (version 6.5, Chap. 6.5.2.8) [34]. For studies providing individual participant data (IPD), means and SD were calculated directly from the raw data of ET patients only. We used plot digitizer for extracting data reported only in graphs [35].

$$SD=\sqrt{N}\times\:\left(Upper\:limit-lower\:limit\right)/\:3.92$$

$${Mean}_{Change}={Mean}_{Final}-{Mean}_{Baseline}\:$$

$${SD}_{Change}=\sqrt{{SD}_{Baseline}^{2}+{SD}_{Final}^{2}-\left(2\times\:r\times\:{SD}_{Baseline}\times\:{SD}_{Final}\right)}$$

The meta-analyses models were performed using a random-effects model, given the clinical heterogeneity across stimulations paradigms, devices, protocols, and follow-up durations, using Review Manager (RevMan) version 5.4.1. For both TETRAS-PS and BF-ADL, the mean change from baseline was pooled as a mean difference (MD) with 95% confidence interval (CI). Statistical heterogeneity was assessed using Cochran’s Q test, with *p* < 0.1 considered indicative of significant heterogeneity and measured using the I² statistic.

### Publication bias

According to Egger et al.‘s paper [36], publication bias requires at least ten papers. Therefore, in our study, we were unable to assess publication bias using Egger’s funnel plot asymmetry.

## Results

### Search results

**Fig. 1:**
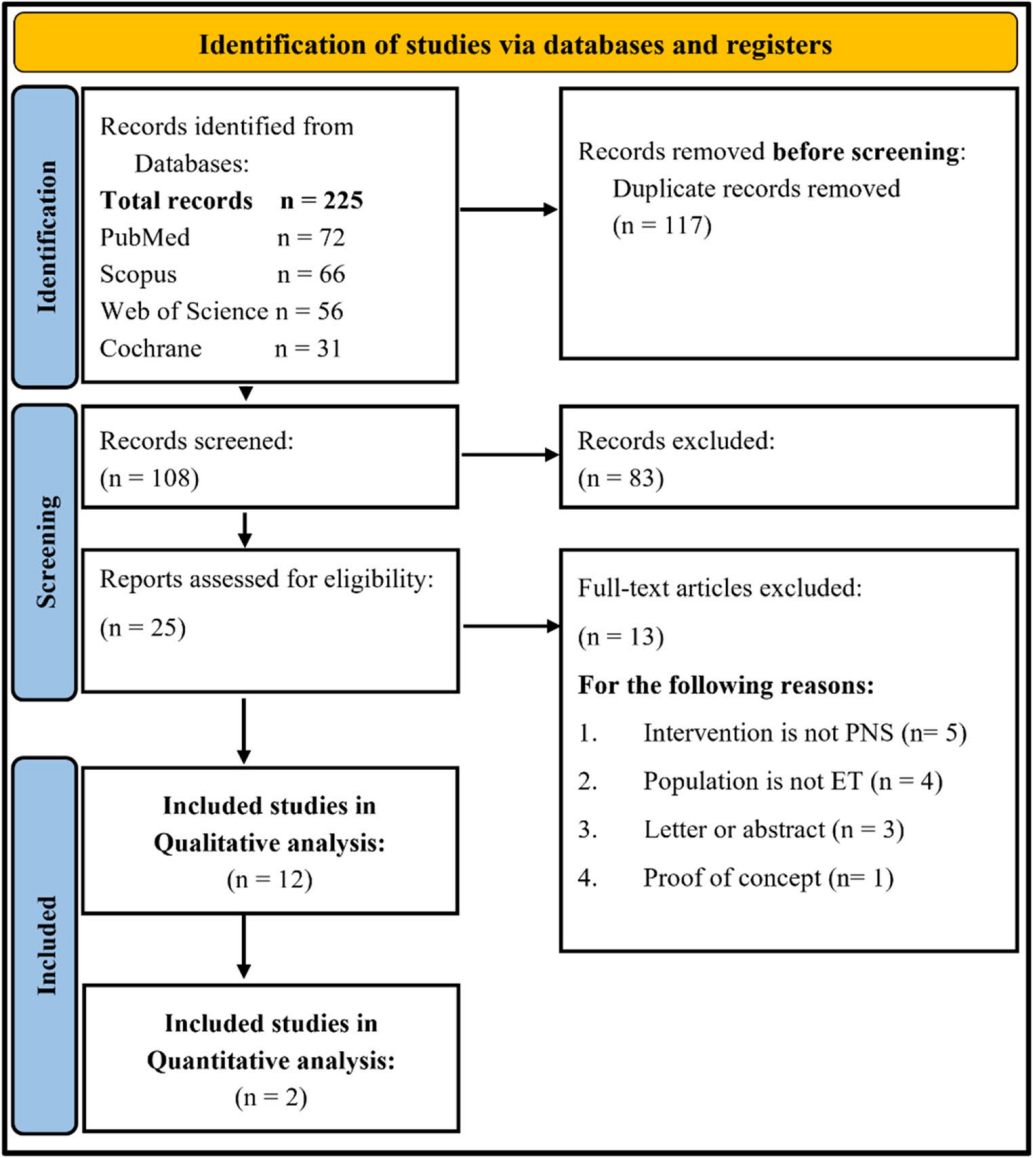
PRISMA flow diagram

Our search retrieved 225 unique studies, of which 117 were duplicates. After manually removing duplicates, 108 studies remained for title and abstract screening. Following this screening, 83 studies were excluded, leaving 25 studies for full-text eligibility assessment. Of the 25 studies, 12 met the inclusion criteria and were included in the review, of which two studies were included in the meta-analysis model. (Fig. 1) presents the PRISMA flow diagram detailing the study identification and selection process.

### Study characteristics

The included studies comprised four RCTs [18, 19, 22, 23] and eight non-randomized studies, including open-label [20, 21, 37, 38], exploratory pre–post [39, 40], and mechanistic studies [41, 42]. Most studies were conducted in the United States (*n* = 8), with one study each from Iran [23], Spain [37], and Canada [40], and one multicenter study conducted in the United States and China [18].

The most frequently investigated intervention was TAPS with or without standard of care (SOC) (*n* = 5), which used alternating stimulation bursts matched to the patient’s tremor frequency measured by an onboard accelerometer. Two studies evaluated AI-TPNS, which used continuous wrist motion recordings analyzed by cloud-based machine-learning algorithms to adjust stimulation parameters in real time [18, 20]. A key difference between traditional closed-loop systems and AI-assisted systems is that closed-loop systems adjust stimulation according to predefined rules based on real-time sensor data; whereas AI-assisted systems continuously learn patterns from patient movement data, analyze them, and then optimize stimulation delivery accordingly [20, 43]. Other approaches included phase-locked stimulation synchronized to specific phases of the tremor cycle using accelerometer-derived signals [39], intramuscular nerve stimulation triggered by electromyographic activity from antagonist muscles [37], and TENS applied over the brachial plexus using fixed stimulation parameters [40] (Table 1).

**Table 1:** Demographic characteristics of included studies

Study ID	Location	Registration number	Study design	Treatment Duration	Evaluation Time Points	Population	Inclusion criteria	Exclusion criteria	Intervention
Dai [19]	US	NCT05540626	RCT	30 days	Baseline & 1 month	310 ET patients	Adults (≥22 years) with ET, identified by ≥2 ICD-10-CM G25.0 claims ≥7 days apart or 1 ET claim plus ET medication, who had ≥12 months of continuous commercial or Medicare Advantage medical and pharmacy coverage and provided informed consent.	patients with neurologic or thyroid disorders, implanted electronic devices, prior ET-related neurosurgery, upper-limb botulinum toxin use within 6 months, current or planned pregnancy, or hand skin lesions at the stimulation site.	TAPS + SOC
Ondo [18]	US & China	NCT06235190	RCT	90 days	Baseline, 14, 30, 60, 90 days	125 ET patients	Adults (≥18 years) with clinically diagnosed ET and upper-extremity tremor, meeting TETRAS severity criteria, able to use a smartphone/Wi-Fi, on no or stable ET medications, with stable caffeine, alcohol, and marijuana use.	Patients with non-ET causes of tremor, upper-extremity conditions interfering with treatment, prior ET surgery, substance use disorder or recreational drug use (except marijuana), adhesive allergy, or prior TAPS use.	AI-TPNS
Pahwa [22]	US	NCT02629614	RCT	1 day	Before and immediately after the end of the session	93 ET patients	Adults (≥22 years) with clinically confirmed ET, informed consent, TETRAS spiral score ≥2 in at least one hand, and BF-ADL score ≥3 on any item.	Patients with implanted electrical devices, prior thalamotomy, seizure disorders, pregnancy, skin lesions at the stimulation site, peripheral neuropathy, alcohol dependence, other tremor causes, inconsistent neurologic exam, or recent alcohol/caffeine use.	PNS
Samiee [23]	Iran	IRCT2016-1212031362N2	RCT	1 day	Baseline and immediately, 1 hr., 3 hr., 4hr, 5hr, 6hr, 24hr after intervention	88 ET patients	Adults with clinically confirmed ET, stable tremor and other medications (if any), insufficient response to tremor medications, and TETRAS score ≥1 or BF-ADL score ≥2 at baseline.	Pregnancy, severe liver/kidney disease, implanted medical devices, other neurological conditions, recent hand botulinum injections or prior thalamotomy, recent alcohol/caffeine use, alcohol dependence or high caffeine intake, forearm implants or compromised skin, and inability or unwillingness to follow the study protocol.	PNS
Dewey [20]	US	NCT05842434	prospective, open-label, pilot study	7 to 10 days	Baseline and after 7 - 10 days	17 ET patients	Adults (≥18 years) with clinically diagnosed ET, TETRAS upper-limb score ≥2 on at least one task and total ≥7, stable medications for ≥30 days, able to use a smartphone and home Wi-Fi, and willing to provide informed consent.	Patients with upper-limb amputation or peripheral neuropathy, substance abuse, recent seizures, pregnancy or planned pregnancy, skin issues at the stimulation site or adhesive allergy, dementia/Alzheimer’s, or recent hand botulinum toxin injections; alcohol and marijuana use were limited, and caffeine intake had to remain stable.	AI-TPNS
Luu [41]	US	IRB ID: 2018 - 1052	Single-arm, exploratory mechanistic study	1 day	Baseline, immediately post-TAPS & Intra-operative during DBS surgery	9 ET patients	Adults (18–85 years) with TETRAS spiral score ≥2 in the dominant hand.	Pregnancy or cognitive impairments affect consent or study participation.	TAPS
Pascual-Valdunciel et al. [37]	Spain	NA	prospective, single arm	2 sessions separated by at least 1 week	Baseline, immediately post-Treatment and 24 hr. post treatment	9 ET patients	Adults (18–80 years) with ET affecting at least one upper limb, no other neurological/musculoskeletal disorders, able to consent.	Other movement-affecting diseases, complex tremors, serious medical conditions, anticoagulant use, or absent tremors at first session.	IMES
Yu [38]	US	NA	Single arm, open label	1 day	baseline, during stimulation, immediately after stimulation, 30 min., 60min. after stimulation	15 ET patients	Patients (≥22 years) with ET diagnosed by a doctor and willing to follow the study protocol.	Patients were excluded for implanted electrical devices, mild tremors, peripheral neuropathy, alcoholism, other known tremor causes, epilepsy, heart rhythm issues, recent tremor medication changes, recent alcohol/caffeine intake, recent clinical trial participation, pregnancy, or inability to communicate.	TAPS
Isaacson [21]	US	NCT03597100	prospective, open-label, post-clearance, single-arm	90 days	Baseline, 1month, 3 months	263 ET patients	Patients (≥22 years) with a physician-diagnosed ET, showing significant tremors on dominant-hand tasks (TETRAS ≥2, total ≥6) and daily activity impact (BF-ADL ≥3 per task, total ≥8).	Patients were excluded for prior DBS or thalamotomy, epilepsy, skin issues at the stimulation site, neuropathy, other neurodegenerative diseases, recent botulinum toxin use, pregnancy, or recent alcohol/caffeine intake.	TAPS
Barath [42]	US	NCT03778060	pilot longitudinal study	90 days	Baseline & 90 days	5 ET patients	Adults (≥21 years) approved for DBS for essential tremors, able to consent, with stable tremor (≥30 days) and antidepressant (≥90 days) medications, if applicable, and willing to comply with protocol requirements. Alcohol/caffeine and Cala Two use were restricted before visits. Pregnancy testing was required for women of child-bearing potential before optional PET/CT, which required full Cala Two study eligibility.	alcohol dependence; implanted electrical devices; prior thalamotomy; seizure disorders; wrist skin pathology; peripheral neuropathy; neurodegenerative disease or Parkinsonism; recent botulinum toxin or trial participation; significant alcohol/caffeine intake; inability to comply; medical contraindications; and pregnancy. PET/CT exclusions were inability to remain still, hyperglycemia, claustrophobia, or failed pregnancy testing.	TAPS
Kim [39]	US	NA	exploratory pre-post design	1 day	Baseline & during intervention	9 ET patients	ET who provided written informed consent and were able to perform the bean-transfer task used to elicit kinetic tremor	NA	PNS
Munhoz [40]	Canada	NA	exploratory pre-post design	1 day	baseline, during intervention, immediately after intervention	5 ET patients	patients able to tolerate TENS stimulation and complete clinical and electrophysiological tremor assessments	NA	TENS
Study ID	Device name	Number of sessions or exposure	Nerves stimulated	Place of intervention	Comparator	Outcome	Key findings	Adverse effects	
Dai [19]	Cala trio	40 min. session (as needed) for 30 days	median & radial	Home	SOC	tremor power, BF-ADL	Adding TAPS to SOC significantly improved tremor severity and BF-ADL scores compared with SOC alone over one month, supporting TAPS as a safe and effective treatment option for ET.	4 patients reported wrist skin irritation, sores, discomfort, or dizziness, all of which were resolved without any professional medical attention, and no SAEs were reported	
Ondo [18]	Felix Neuro AI Wristband	Continuous use during waking hours	Radial, Median & Ulnar	Home	Sham	mADL subscale of TETRAS, CGI-I, CGI-S, PGI-I, PGI-S, QUEST	The TPNS device improved activities related to the upper limbtremor at 90 days and could be an effective noninvasive ET treatment.	Adverse events occurred on 28/83 (33.7%) TPNS patients vs 2/42 (4.8%) sham. Skin irritation was most common (24/83; 28.9% mild, 3/83; 3.6% moderate), while 2/42 (4.8%) sham patients had moderate irritation. Other isolated AEs (1/83; 1.2% each, TPNS only) included nausea, arthralgia, thumb arthritis worsening, muscular weakness, limb discomfort, and involuntary muscle contractions; no serious AEs were reported.	
Pahwa [22]	cala ONE	40 min. single session	Median & Radial	Clinic	Sham	TETRAS Archimedes spiral score, TETRAS upper limb tremor score, BF-ADL, CGI-I	PNS in ET may provide safe, well-tolerated, and effective treatment for transient reliefof hand tremor symptoms.	a low adverse event rate (3%), with mild, short-lived effects such as skin irritation or wrist discomfort that resolved within 24 hours. No serious or unanticipated device-related events occurred, and no participants reduced or stopped stimulation.	
Samiee [23]	NA	40 min. single session	Median & Radial nerve	Hospital	Sham	Tremor amplitude, TETRAS, BF-ADL, CGI-I	PNS significantly reduced tremor amplitude compared to sham, but no differences were seen in TETRAS, BF-ADL, or CGI-I scores, possibly due to placebo effects, single-session design, or questionnaire validation issues.	One participant in the non-intervention group experienced mild, transient skin irritation (redness, itching).self-resolved within 15–20 min. No other adverse events were observed.	
Dewey [20]	FelixTM Neuro AI TM Wrist band	During waking hours	Radial, Median & Ulnar	Home	NA	TETRAS PS, TETRAS ADL, mADL, PGI-I, CGI-I	In an uncontrolled pilot study, AI-TPNS, worn for 7–10 days, significantly reduced tremor in ET patients, with minimal side effects.	One patient developed a mild skin reaction due to a pre-existing adhesive allergy, was withdrawn, and the study added adhesive allergy as an exclusion criterion; the device was modified to minimize skin reaction, and no further adverse events occurred.	
Luu [41]	Cala TWO	1 pre-operative (40min.) & 1 intraoperative	Median & Radial	Hospital	NA	TETRAS – PS (upper-limb), fTR, VIM thalamic local field potentials (LFPs), VIM multi-unit spiking activity	TAPS likely reduces tremor in ET by modulating the VIM and connected nodes in thecerebello-thalamo-cortical pathway.	No adverse events were reported.	
Pascual-Valdunciel et al. [37]	NA	2 sessions	Median & radial nerves (surface stimulation); muscle afferents of FCR & ECR (intramuscular stimulation)	Hospital	NA	FTM, CGI-S, CGI-C, kinetic tremor assessment	both immediate and 24-hour tremor reduction in ET patients is possible using a minimally invasive SATS approach targeting wrist muscle sensory afferents, suggesting a potential new therapy for tremor.	Sessions were well tolerated; minor, brief effects included pain (7 patients) from electrode insertion/removal, paresthesia (2), and fatigue (1).	
Yu [38]	Cala Health device	40min.	Median & Radial	Clinic	NA	FTM-CRS tasks (Spiral drawing, postural hold, Finger-to-nose reach, and pouring)	TAPS therapy is safe and provides lasting tremor reduction for at least 60 minutes, and sensor data can help monitor treatment response at home.	No adverse events were reported.	
Isaacson [21]	Cala Health device	twice daily for 3 months for 40min.	Median & Radial	Home	NA	TETRAS, BF-ADL, CGI-I, PGI-I, QUEST	non-invasive neuromodulation therapy used repeatedly at home overthree months results in safe and effective hand tremor reduction in many essential tremor patients.	skin irritation (5.3%), sore/lesion (3.8%), discomfort (2.3%), electrical burns (2.3%); others ≤1.1% (shock sensation, tremor worsening). Mostly mild – moderate; No serious adverse events reported	
Barath [42]	Cala Health device	40min. session twice daily for 90 days	Median & Radial	Home	NA	TETRAS, brain metabolic changes	TAPS reduces hand tremor in essential tremors. Longitudinal TAPS therapy alters cere-bellar metabolism, which can be a cause or consequence of tremor reduction. Cerebellar-premotor regionconnectivity may play a role in the anti-tremor effects of TAPS.	No adverse events were reported.	
Kim [39]	NA	1 session	radial	Academic research sitting	NA	Tremor frequency, tremor power, TETRAS	Phase-locked radial nerve stimulation reduced tremor power by up to ~60–65%, especially in participants with higher baseline TETRAS scores, using higher amplitudes and out-of-phase (±peak) stimulation, without changing tremor frequency.	No adverse events were reported.	
Munhoz [40]	Selectra, Dual Channel TENS 7720	15min. session	brachial plexus	Hospital	NA	WHIGET rating scale	Acute transcutaneous electrical nerve stimulation did not significantly reduce tremor severity, with no meaningful changes in WHIGET or objective measures. Minor individual or subjective improvements were inconsistent and not significant.	No adverse events were reported.	

*NA* Not applicable, *RCT* Randomized Controlled Trial, *ET* Essential Tremors, *PNS* Peripheral Nerve Stimulation, *TAPS* Transcutaneous afferent patterned stimulation, *SOC* Standard of Care, *TPNS* Transcutaneous peripheral nerve stimulation, *IMES* Intramuscular Electrical Stimulation, *BF-ADL* Bain & Findley Activities of Daily Living, *CGI-I* Clinical Global Impression–Improvement, *CGI-S* Clinical Global Impression–Severity, *CGI-C* Clinical Global Impression–Change, *PGI-I* Patient Global Impression–Improvement, *PGI-S* Patient Global Impression–Severity, *QUEST* Quality of Life in Essential Tremor Questionnaire, *TETRAS* Tremor Research Group Essential Tremor Rating Assessment Scale, *TETRAS PS TETRAS Performance Subscale* TETRAS ADL TETRAS Activities of Daily Living Subscale, *mADL* Modified Activities of Daily Living, *FTM* Fahn–Tolosa–Marín Tremor Rating Scale, *FTM-CRS* Fahn–Tolosa–Marín Clinical Rating Scale, *VIM* Ventral Intermediate Nucleus, *LFPs* Local Field Potentials, *WHIGET* Washington Heights–Inwood Genetic Study of Essential Tremor rating scale, *fTR* Functional Tremor Rating, *FCR* Flexor Carpi Radialis, *ECR* Extensor Carpi Radialis

Across the included studies, interventions were most frequently conducted in home-based settings (*n* = 5), followed by hospital settings (*n* = 4) and clinic-based settings (*n* = 2). Sample sizes ranged from five patients [42] to 263 [21], and treatment duration ranged from one day (*n* = 6) to 90 days (*n* = 3).

A total of 914 participants were included across the studies, with a mean age ranging from 57.7 to 72.2 years and a male prevalence ranging from 40% to 78%. At baseline, mean TETRAS scores ranged from 1.6 [41] to 25.8 [22], while the BF-ADL scores ranged from 2.15 [19] to as high as 45.8 [22] (Table 2).

**Table 2:** baseline characteristics of included studies

Study ID	RCTs					
	Group	Sample size	Age	Gender (Male)	TETRAS PS	BF-ADL
		*N*	Mean (SD)	*N* (%)	Mean (SD)	Mean (SD)
Dai [19]	TAPS + SOC	133	67.77 (11.71)	84 (63.16)	NA	2.16 (0.63)
	SOC	143	68.61 (10.51)	99 (69.23)	NA	2.15 (0.61)
Ondo [18]	AI-TPNS	83	63.8 (13.9)	39 (47)	12.3 (3.1)	NA
	Sham	42	67.2 (11.4)	24 (57.1)	12.9 (3.1)	NA
Pahwa [22]	PNS	48	70.5 (11.2)	23 (48)	25.8 (6)	45.8 (9)
	Sham	45	69.8 (10.1)	22 (49)	24.8 (6)	45 (10.3)
Samiee [23]	PNS	45	65 (10.72)	30 (66.67)	11.11 (5.53)	15.27 (5.89)
	Sham	43	57.67 (15.34)	21 (48.84)	9.86 (5.20)	14.02 (4.95)
	**Observational Studies**					
Study ID	**Group**	**Sample size**	**Age**	**Gender (Male)**	**TETRAS PS**	**BF-ADL**
		**N**	**Mean (SD)**	**N (%)**	**Mean (SD)**	**Mean (SD)**
Dewey [20]	AI-TPNS	17	70.4 (8.1)	9 (52.94)	14.1 (2.2)	NA
Luu [41]	TAPS	9	65 (7)	7 (78)	1.6 (0.4)	NA
Pascual-Valdunciel [37]	IMES	9	70.3 (5.45)	5 (55.56)	44.67 (9.76)^a^	NA
Yu [38]	TAPS	15	72.2 (8.6)	9 (60)	NA	NA
Isaacson [21]	TAPS	263	69.6 (10.1)	126 (47.91)	12.6 (2.7)	18.4 (3.8)
Barath [42]	TAPS	5	70.2 (5.2)	3 (60%)	6.5 (2.5)	NA
Kim [39]	PNS	9	67.67 (11.67)	4 (44.44)	NA	NA
Munhoz [40]*	TENS	5	70 (3.79)	2 (40%)	15.4 (1.96)^b^	NA

*Extracted data of ET patients only; ^a^Total FTM; ^b^WHIGET scale, *SD* Standard Deviation, *NA* Not Applicable, *PNS* Peripheral Nerve Stimulation, *TAPS* Transcutaneous afferent patterned stimulation, *SOC* Standard of Care, *TPNS* Transcutaneous peripheral nerve stimulation, *IMES* Intramuscular Electrical Stimulation

### Assessment of bias and certainty of evidence

**Fig. 2:**
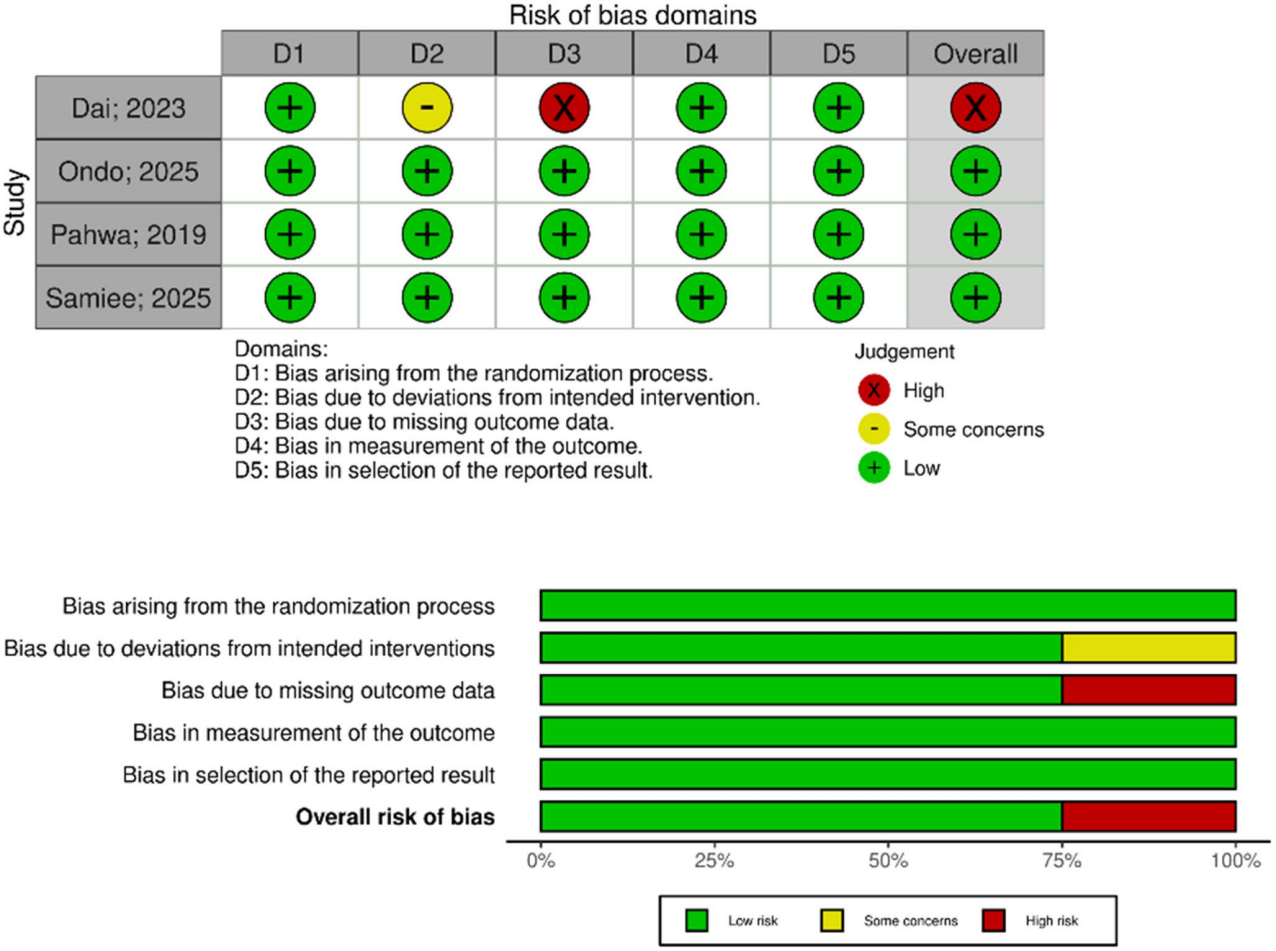
Risk of bias (RoB) 2 assessment of RCTs

Most included RCTs [18, 22, 23] were rated as low risk across all domains. In contrast, Dai et al. [19] was judged as high risk of bias due to significant missing outcome data and some concerns noted in deviations from intended interventions due to the open-label nature of the pragmatic study design (Fig. 2). Regarding the non-randomized studies, only one study [21] was rated as good quality, while the remaining single-arm studies were rated as fair quality, with common limitations including small sample sizes, unblinded outcome assessment, and the absence of repeated pre- and post-intervention measurements (Table 3). Using the GRADE framework, the certainty of the evidence was rated as low for both the TETRAS-PS and BF-ADL scales due to significant imprecision, despite the marked improvements with PNS compared to sham. For the CGI-I scale, certainty was very low due to the high risk of bias and very serious imprecision (Table 4).

**Table 3:** NIH single arm quality assessment

	Scale Items												
	1	2	3	4	5	6	7	8	9	10	11	12	Score
Dewey et al. [20]	Y	Y	Y	Y	N	Y	Y	N	N	N	NA	Y	**Fair Quality**
Luu et al. [41]	Y	Y	Y	Y	N	Y	Y	N	Y	N	NA	Y	**Fair Quality**
Pascual-Valdunciel et al. [37]	Y	Y	Y	Y	N	Y	Y	N	Y	N	NA	Y	**Fair Quality**
Yu et al. [38]	Y	Y	Y	Y	N	Y	Y	N	Y	N	NA	Y	**Fair Quality**
Isaacson et al. [21]	Y	Y	Y	Y	Y	Y	Y	N	Y	N	NA	Y	**High Quality**
Barath [42]	Y	Y	Y	Y	N	Y	Y	N	Y	N	NA	Y	**Fair Quality**
Kim [39]	Y	N	Y	Y	N	Y	Y	N	Y	N	NA	Y	**Fair Quality**
Munhoz [40]	Y	N	Y	Y	N	Y	Y	N	Y	N	NA	Y	**Fair Quality**

NIH tool for pre and post studies without control arm. Q1: Objective clearly stated; Q2: eligibility criteria described; Q3: representative patient population; Q4: all eligible participants enrolled in study; Q5: sufficient sample size; Q6: intervention described; Q7: outcome measures specified; Q8: outcome assessors blinded; Q9: loss to follow−up; Q10: statistical analysis of outcome measures before and after intervention; Q11: interrupted time−series design; Q12: individual data used for group−level effects. *Y* yes, *N* no, *NA* not applicable

**Table 4:** Summary of findings regarding certainty judgment using GRADE framework

Certainty assessment							№ of patients		Effect	Certainty
№ of studies	Study design	Risk of bias	Inconsistency	Indirectness	Imprecision	Other considerations	PNS	Sham treatment	Absolute (95% CI)	
TETRAS-PS*										
2	RCTs	not serious	not serious	not serious	very serious^a, b,c^	none	85	80	MD= -0.92, 95% CI [-1.63 to -0.21]	⨁⨁◯◯ Low^a, b,c^
BF-ADL*										
2	RCTs	not serious	not serious	not serious	very serious^a, b,c^	none	85	80	MD = -1.90, 95%CI [-2.97 to -0.83]	⨁⨁◯◯ Low^a, b,c^
CGI-I**										
5	3 RCTs and 2 Observational Studies	serious^d, e^	not serious	not serious	very serious^f, g^	none	-			⨁◯◯◯ Very low^d, e,f, g^

*GRADE assessment based on meta-analysis mode; **GRADE assessment based on narrative synthesis only; ^a^only 2 studies added to the meta-analysis model; ^b^Pahwa et al. contributed to most of the weight realtive to Smaiee et al.; ^c^small number of participants in both intervention and control groups; ^d^CGI-I is a subjective clinician-rated outcome.; ^e^PNS often causes paresthesia, potentially compromising blinding.; ^f^CGI-I was narratively synthesized, so no pooled effect estimate.; ^g^Small-to-moderate sample sizes.; *TETRAS-PS* Tremor Research Group Essential Tremor Rating Assessment Scale - Performance Subscale, *BF-ADL* Bain & Findley Activities of Daily Living, *CGI-I* Clinical Global Impression–Improvement, *CI* confidence interval, *MD* mean difference, *PNS* Peripheral Nerve Stimulation, *RCTs* Randomized Controlled trial

### Mechanistic evidence underlying tremor suppression

Two studies primarily investigated the underlying mechanisms of tremor suppression following PNS: Barath et al. and Luu et al. studies [41, 42]. Barath et al. examined longitudinal changes in brain metabolism following TAPS using positron emission tomography (PET) imaging [42]. After 90 days of TAPS, significant alterations in cerebellar metabolism were observed, which correlated with improvements in clinical tremor scores (*r* = − 0.70 and − 0.56). Although, the study recorded a highly significant 72.6% median reduction in objective tremor power (*p* < 0.0001), the cumulative reduction in baseline tremor scores did not reach statistical significance (*p* = 0.14).

Luu et al. tried to investigate the underlying mechanism of TAPS in nine ET patients undergoing DBS surgery using intraoperative microelectrode recordings [41]. They observed that the tremor reduction was positively correlated with TAPS with modulation of alpha-band (R2 = 0.213, *p* < 0.001) and beta-band (R2 = 0.255, *p* < 0.001) local field potentials (LFPs) in the ventral intermediate nucleus (VIM). Additionally, TAPS suppressed spiking activity in the VIM (R2 = 0.104, *p* = 0.029); however, it did not correlate with degree of tremor reduction. Collectively, both mechanistic studies suggest that PNS may exert therapeutic effects through central neural networks modulation besides its peripheral activity.

### Exploratory or proof-of-concept studies

Three studies explored alternative paradigms and their potential in managing the tremors in ET patients. Dewey et al. showed a significant reduction (*p* = 0.0002) in tremors in 17 ET patients managed using AI-assisted TPNS device as TETRAS-PS scale went from 14.1 at baseline to 11.4 after 7 to 10 days follow up. Additionally, a significant improvement in ADL of these patients has been observed (*p* < 0.0001) [20].

Kim et al. explored the effect of different stimulation parameters, including different amplitudes (low, medium, and high), different frequencies (100 and 200 Hz), and different stimulation phases synchronized to tremor cycle in 9 ET patients [39]. Stimulation significantly reduced tremor power at all amplitudes (all *p* < 0.001), while its effect on tremor frequency was minimal (*p* = 0.134, 0.086, and 0.036 for low, medium, and high amplitude stimulation, respectively). Additionally, higher stimulation amplitudes resulted in greater tremor power reduction up to 65% with patients exhibiting a more severe baseline tremor. Tremor suppression was significantly affected by both stimulation amplitude and phase (all p-values < 0.001), with out-of-phase stimulation (½π) providing the greatest benefit. A significant decrease in tremor power at the highest stimulation amplitude was observed in patients with a TETRAS score of 3 (*p* = 0.027), while patients with a TETRAS score of 4 showed significant differences between low and medium amplitude stimulation (*p* = 0.046) and greater tremor suppression with ½π phase compared to π phase stimulation (*p* = 0.039).

Pascual-Valdunciel et al. assessed a minimally invasive intramuscular stimulation system targeting muscle afferents was evaluated in 9 patients with ET [37]. Selective and adaptive timely stimulation (SATS) through the intramuscular electrodes resulted in an approximately 32% reduction in tremors; however, continuous stimulation led to increased tremorgenic activity. Furthermore, the tremor reduction was greater with intramuscular stimulation compared to surface stimulation (*p* < 0.05), with the reduction persisting for up to 24 h post-stimulation in four patients.

### Efficacy outcomes

Only two trials were included in the quantitative meta-analysis because they alone reported the same outcomes (TETRAS-PS and BF-ADL) immediately post-stimulation [22, 23]. The other RCTs used different treatment durations and follow-ups (e.g., tremor power or mADL at 1–3 months) [18, 19]. The remaining eight studies were single-arm, mechanistic, or pilot designs that either lacked a control arm or did not provide the required summary statistics. These inconsistencies in outcome measures, timing, and study designs prevented pooling, so the meta-analysis was restricted to the two directly comparable trials.

#### Immediate post-stimulation effects

**Fig. 3:**
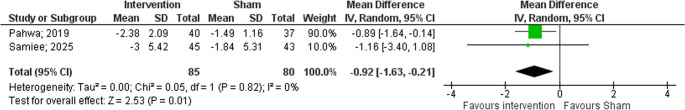
Forest plots of the mean difference (MD) of TETRAS-PS immediate post-stimulation. IV, inverse variance; CI, confidence interval

Regarding clinician-rated TETRAS immediately post-stimulation, the pooling two randomized trials with 80 patients revealed a significant reduction in clinician-rated tremor severity following a single stimulation session (MD= -0.92, 95% CI [-1.63 to -0.21], *p* = 0.01, I^2^ = 0) (Fig. 3). There was no significant heterogeneity, although the pooled estimate is driven primarily by the trial reporting larger effects. At the trial level, Pahwa et al. [22] found a significantly greater reduction in clinically rated Upper Limb Tremor scores for the active treatment group compared to sham (42% vs. 28%), whereas Samiee et al. [23] observed no statistically significant differences between groups. Moreover, both studies noted significantly better clinician-rated CGI-I scores in the intervention group [22, 23].

**Fig. 4:**
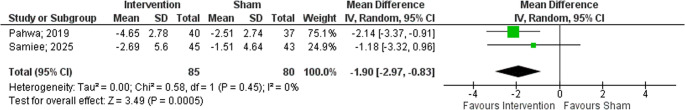
Forest plots of the mean difference (MD) of BF-ADL immediate post-stimulation. IV, inverse variance; CI, confidence interval

A similar pattern was observed for patient-reported functional outcomes. The meta-analysis demonstrated a significant improvement in BF-ADL (MD = -1.90, 95%CI [-2.97 to -0.83], *p* = 0.0005, I^2^ = 0), without any observed heterogeneity (Fig. 4). Individually, Pahwa et al. [22] reported a 49% reduction in BF-ADL scores for the active treatment group versus 27% for sham, whereas Samiee et al. [23] found no statistically significant differences between groups.

Regarding the effect of stimulation on objective tremor power, in their RCT comparing TAPS to SOC, Dai et al. [19] found that median tremor power decreased significantly from 0.038 to 0.017 (m/s²)² post-stimulation (*p* < 0.0001). Similarly, in their single-arm studies, Yu et al. observed a 70% reduction in postural hold tremor power immediately after stimulation [38], while Isaacson et al. reported that 54% of participants experienced ≥ 50% improvement in tremor power [21]. However, results for spiral drawing, an objective task-based measure of tremor, were mixed as Pahwa et al. [22] found no significant difference, whereas Luu et al. reported improvement in the treated hand following a single TAPS session in their single-arm, exploratory mechanistic study.

Regarding the duration of acute effects, over 70% of participants in a single-arm, open-label study maintained improved tremor power for at least 60 min [38]. While Samiee et al. [23] and Isaacson et al. [21] observed sustained improvements up to 90 min post-stimulation.

#### Outcomes after one month of continuous treatment

Three studies evaluated outcomes after one month, with the active stimulation treatment protocol continuing throughout this duration. In their randomized trial, Dai et al. [19] reported that median tremor power was markedly lower with TAPS plus SOC than with SOC alone (0.017 vs. 0.08 (m/s²)²; *p* < 0.0001), with a sustained reduction in baseline tremor power by one month (*p* = 0.0018). Dai et al. [19] also reported that BF-ADL scores improved more in the TAPS group than SOC alone (mean change 1.6 vs. 0.2 points, *p* < 0.05).

Similarly, a single-arm study reported a 50% reduction in tremor power during the first month [21]. Furthermore, clinician-rated TETRAS scores improved from 12.6 at baseline to 9.7 immediately post-stimulation, and further to 11.9 after one month. while patient-reported BF-ADL scores also improved, decreasing from 18.4 at baseline to 16.4 just post-stimulation and 13.7 post-stimulation at one month [21].

Meanwhile, in the sham-controlled trial by Ondo et al. [18], the AI-TPNS trended toward improvement in modified activities of daily living (mADL) scores after one month of use, although between-group differences had not reached statistical significance (*p* = 0.08).

#### Outcomes after three months of continuous treatment

Three studies evaluated outcomes after a three-month (90 days) treatment duration. Ondo et al. [18] reported that AI-TPNS produced significantly greater reduction in mADL scores than sham (mean change − 6.9 vs. − 2.7; *p* < 0.001). CGI-I was observed in 69.4% of patients (*p* = 0.02), while 68% of patients self-reported improvement on the Patient Global Impression–Improvement (PGI-I) (*p* = 0.04). The intervention also led to clinically meaningful gains in multiple in the Quality of Life in Essential Tremor Questionnaire (QUEST) domains.

Similarly, Isaacson et al. [21] reported significant improvements from baseline to three months in both clinician-rated TETRAS and patient-reported BF-ADL scores (*p* < 0.0001), with 62% of patients improving from moderate/severe to mild/slight tremors. Further, tremor power improved in 92% of participants, alongside favorable clinician (68%) and patient (60%) global impressions.

### Safety and tolerability

Across included studies, TPNS/TAPS demonstrated a favorable safety and tolerability profile, with no serious device-related adverse events reported across studies. In long-term studies, skin-related adverse events were more frequent with active stimulation than with sham but remained mild. For instance, Ondo et al. [18] reported skin irritation in 33.7% of active TPNS participants versus 4.8% in the sham group, while Isaacson et al. [21] reported device-related adverse events in 18% of participants, mainly persistent skin irritation (5%), sores or lesions (4%), and electrical burns (2%). Dai et al. [19] reported temporary wrist irritation or discomfort in 3% of participants over one month. Acute and short-term studies reported very low adverse-event rates, limited to mild transient skin or sensory symptoms [20, 22, 23], with one study reporting no adverse events [38]. Withdrawals due to adverse events were uncommon (≤ 5%) [18, 21]. All adverse effects with details are provided in (Table 1).

## Discussion

The synthesis of evidence in this review indicates that non-invasive PNS may be associated with modest reductions in tremor severity and improvement in functional performance in patients with ET; however, to the best of our knowledge, the minimum clinically important difference (MCID) in these measures has not been established, limiting the interpretation of these findings and leaving their clinical significance uncertain.

Although our meta-analysis is confined to only two trials, these pooled estimates are consistent with objective tremor-power reductions reported in other several trials. Furthermore, longitudinal data from home-use studies, although limited to up to three months, suggest that these effects may persist over extended periods post-stimulation, with a notable proportion of patients experiencing a transition to milder symptoms. Notably, non-invasive PNS was associated with mainly mild, local skin reactions, highlighting its favorable safety profile.

While the pooled meta-analysis demonstrated a significant reduction in clinician-rated tremor severity and in patient-reported functional disability, clinical heterogeneity in the results of the included trials warrant consideration. Pahwa et al. [22] reported significant improvements on clinician- and patient-rated scales, whereas Samiee et al. [23] found no significant changes on TETRAS or BF-ADL. This discrepancy is likely due to baseline differences: Pahwa et al.’s cohort had higher tremor severity (mean TETRAS = 25.8 (6)), allowing greater detectable change, while Samiee et al.’s cohort had milder tremor (mean TETRAS = 11.11 (5.53)), limiting the sensitivity of ordinal scales. Notably, Samiee et al. observed a significant reduction in objective tremor amplitude measured via accelerometry [23]. These findings underscore that, in populations with mild ET, objective kinematic measures may be necessary to accurately assess PNS efficacy, as traditional clinical scales are prone to floor effects [44]. Crucially, the challenge of maintaining effective blinding in PNS trials complicates the interpretation of these subjective scales. Because active stimulation produces perceptible paresthesia, both expected and placebo responses may contribute to observed benefits, particularly in patient-reported measures. Notably, Pahwa et al. implemented specific measures to maintain masking, including informing participants that they “may or may not feel stimulation” and exposing both the intervention and control groups to sensory stimulation during device calibration, demonstrating the investigators’ awareness of potential sensory unblinding [22]. Consequently, a meaningful portion of the observed benefit on patient-reported measures may reflect a sensory placebo rather than a true therapeutic effect. Our pooled data align with this concern, as the effect size for the patient-reported BF-ADL is proportionally larger than that for the clinician-rated TETRAS-PS (MD = -1.90 and − 0.92, respectively). Nevertheless, the broader clinical efficacy of PNS is supported by other trials, with consistent improvements in clinician-rated TETRAS in open-label cohorts [20, 21] and enhancements in activities of daily living reported across multiple large-scale and home-use studies [18, 19].

The observed reduction in tremor caused by PNS is likely mediated by the modulation of the CTC network, the primary circuit implicated in the pathophysiology of ET [45]. How non-invasive wrist stimulation can disrupt the pathological central oscillations underlying ET may be explained by sustained peripheral stimulation that alters motor cortex excitability through cerebellar mechanisms [46]. This hypothesis is supported by Luu et al., who reported that PNS modulated neuronal spiking activity in the VIM nucleus of the thalamus [41]. Clinically, this short-term network desynchronization is reflected in a transient post-stimulation effect, with studies reporting that tremor suppression remains significant for approximately an hour after stimulation ceases [23, 38]. Furthermore, with chronic use, these acute excitability changes may evolve into sustained network adaptations by significant metabolic changes in the cerebellum and thalamus [42].

In the context of current ET management, where pharmacotherapy often provides limited benefit [47], PNS appears to offer several advantages. Current first-line agents such as propranolol and primidone achieve a positive response in only 30–60% of patients [48], and in these patients, the tremor reduction is only 40%–60% [49] with frequent discontinuation due to adverse effects [50]. In contrast, post-market surveillance data show sustained PNS use for up to three years, with a mean of 5.6 sessions per week, suggesting good real-world tolerability [51, 52]. Although tremor reduction with PNS (~ 50–60% [21, 38]) is lower than that reported for surgical interventions such as DBS or MRgFUS (> 80%) [53], direct efficacy comparisons should be interpreted cautiously. Specifically, surgical cohorts typically consist of severe, medically refractory patients evaluated over multi-year periods, whereas the current PNS trials encompass a broader ET population with significantly shorter follow-up durations. Nevertheless, despite these methodological differences, PNS offers a distinct clinical advantage by avoiding the procedure-related risks associated with invasive interventions, such as persistent gait disturbance and paresthesia [54]. Importantly, PNS may also mitigate inequities in tremor care, as studies show that access to surgical therapies remains disproportionately limited among racial minorities, women, and individuals of lower socioeconomic status due to reliance on specialized tertiary centers [55, 56] while PNS shows significant results at home [18, 19, 21]. As such, PNS may occupy a potentially relevant position between sub-optimally effective pharmacotherapy and invasive or inaccessible surgical options.

It is important to distinguish the stimulation approaches employed in recent trials from earlier peripheral neuromodulation approaches. Previous studies utilizing standard open-loop TENS failed to demonstrate significant tremor suppression [40]. In contrast, more recent interventions have incorporated patient-specific stimulation paradigms tailored to individual tremor characteristics. For example, Kim et al. found that phase-locked stimulation synchronized to the tremor cycle produces greater suppression than continuous open-loop stimulation [39]. Additionally, some recent trials have utilized closed-loop systems, which make use of cloud-based artificial intelligence to continuously adjust stimulation parameters in real-time based on the patient’s tremor fluctuations [18, 20]. This adaptive approach is hypothesized to prevent neural habituation, thereby maintaining therapeutic efficacy throughout the day [57]. Nevertheless, the current evidence remains limited, and further comparative studies are needed to determine their contribution to clinical outcomes.

Beyond efficacy, the safety profile of PNS appears favorable. Across the randomized trials included, no device-related serious adverse events were reported, with adverse effects largely limited to transient skin irritation at the stimulation site [18, 21]. This contrasts with the systemic adverse effects of pharmacotherapy, which frequently lead to treatment discontinuation [47, 50]. In addition, the non-invasive nature of PNS permits on-demand use with sessions scheduled around specific functional tasks, allowing targeted symptom control that is not achievable with fixed pharmacologic dosing or irreversible surgical interventions [52].

## Limitations and recommendations

Despite the current evidence supporting the acute efficacy of PNS in the treatment of ET, several gaps remain. First, the durability of benefit beyond three months is unclear, in contrast to surgical interventions with multi-year follow-up data [58]. Furthermore, much of the available long-term evidence relies heavily on open-label, single-arm data. Future studies should employ longitudinal randomized designs with follow-up exceeding one year to determine whether treatment tolerance or habituation occurs.

Second, there is considerable heterogeneity across the included studies regarding device specifications (e.g., open-loop versus closed-loop AI-driven systems), stimulation parameters, and treatment protocols. This variability complicates the direct comparison of results across trials and limits the ability to identify optimal, standardized therapeutic regimens.

Third, the assessment of efficacy is hindered by outcome variability and the mixed use of objective kinematic metrics (e.g., accelerometry) versus subjective clinical and patient-reported scales. This reliance on subjective measures is particularly problematic given the challenge of maintaining effective blinding in device trials, as active stimulation produces perceptible paresthesia [23, 38, 40, 41]. To mitigate this bias, future trials should prioritize objective endpoints and incorporate improved sham designs, such as active sham stimulation using non-therapeutic frequencies, to better distinguish physiological effects from sensory placebo responses.

Finally, the therapeutic scope of PNS should be evaluated across different tremor phenotypes. Current wrist-worn systems primarily target appendicular tremors via median and radial nerve stimulation, with limited evidence for efficacy in axial symptoms such as head or voice tremors. Given that pharmacotherapy and DBS address these midline manifestations, future technological developments should explore alternative stimulation targets or configurations to extend applicability to axial-predominant disease. In addition, the findings of our meta-analysis should be interpreted with caution, as they are based on only two trials. Consequently, the pooled estimates are influenced substantially by Pahwa et al. study which weighs about 90% and 75.1% for TETRAS-PS and BF-ADL outcomes respectively relative to Samiee et al. study. As a result, this limits the generalizability and strength of our findings despite their consistency with several other trials.

### Conclusion

This systematic review and meta-analysis suggest that non-invasive PNS provides modest reductions in clinician-rated tremor severity and improves patient-reported function in ET, with evidence reaching up to three months. The intervention appears to be tolerable, with mainly mild, local skin reactions, and meaningful improvements in activities of daily living. These findings support the potential of PNS to be integrated as a therapeutic option into the management of ET, alongside established pharmacological and surgical interventions. Nevertheless, further studies with longer follow-up and better randomization protocols are warranted to confirm their efficacy, define durability of benefit, patient subgroups most likely to respond, and clarify the comparative role of PNS relative to pharmacotherapy and surgical options.

## Supplementary Information

Below is the link to the electronic supplementary material.

Supplementary Material 1 (DOCX 1.58 MB)

## Data Availability

All data generated or analyzed during this study are included in this published article and its supplementary information files.

## Abbreviations

AI: Artificial Intelligence
BF-ADL: Bain & Findley Activity of Daily Living
CGI-I: Clinical Global Impression–Improvement
CTC: Cerebello-Thalamo-Cortical
DBS: Deep Brain Stimulation
ET: Essential Tremors
FTM: Fahn–Tolosa–Marin Scale
LFPs: Local Field Potentials
mADL: modified Activities of Daily Living
MCID: Minimal Clinical Important Difference
MRgFUS: Magnetic Resonance-guided Focused Ultrasound
PNS: Peripheral Nerve Stimulation
QUEST: Quality of Life in Essential Tremor Questionnaire
SOC: Standard of Care
TENS: Transcutaneous Electrical Nerve Stimulation
TETRAS: Tremor Research Group Essential Tremor Rating Assessment Scale
TETRAS - PS: Tremor Research Group Essential Tremor Rating Assessment - Performance Scale
VIM nucleus: Ventral Intermediate nucleus
WHIGET: Washington Heights-Inwood Genetic Study of Essential Tremor Rating Scale

## References

[1] Louis ED, McCreary M (2021) How common is essential tremor? Update on the worldwide prevalence of essential tremor. Tremor Other Hyperkinetic Movements 11. 10.5334/tohm.632 10.5334/tohm.632 PMC8269764 34277141

[2] Koller W, Biary N, Cone S (1986) Disability in essential tremor: effect of treatment. Neurology 36:1001–1004. 10.1212/wnl.36.7.1001 2940473 10.1212/wnl.36.7.1001

[3] Thach WT (1978) Correlation of neural discharge with pattern and force of muscular activity, joint position, and direction of intended next movement in motor cortex and cerebellum. J Neurophysiol 41:654–676. 10.1152/jn.1978.41.3.654 96223 10.1152/jn.1978.41.3.654

[4] Dum RP, Strick PL (2003) An Unfolded Map of the Cerebellar Dentate Nucleus and its Projections to the Cerebral Cortex. J Neurophysiol 89:634–639. 10.1152/jn.00626.2002 12522208 10.1152/jn.00626.2002

[5] Suminski AJ, Doudlah RC, Scheidt RA (2022) Neural correlates of multisensory integration for feedback stabilization of the wrist. Front Integr Neurosci 16. 10.3389/fnint.2022.815750 10.3389/fnint.2022.815750 PMC9121119 35600223

[6] Dai D, Samiian A, Fernandes J, Coetzer H (2022) Multiple comorbidities, psychiatric disorders, healthcare resource utilization and costs among adults with essential tremor: a retrospective observational study in a large US commercially insured and medicare advantage population. JHEOR 9. 10.36469/001c.37307 10.36469/001c.37307 PMC9378814 36051002

[7] Zesiewicz TA, Elble RJ, Louis ED et al (2011) Evidence-based guideline update: Treatment of essential tremor. Neurology 77:1752–1755. 10.1212/WNL.0b013e318236f0fd 22013182 10.1212/WNL.0b013e318236f0fd PMC3208950

[8] Update Treatment of Essential Tremor. https://www.aan.com/Guidelines/home/GuidelineDetail/492/utm. Accessed 27 Jan 2026

[9] Gerbasi M, Tu L, Chertavian E et al (2024) Real-world Evidence of Efficacy, Use, and Discontinuation of Pharmacotherapies for the Treatment of Essential Tremor (P3-3.006). Neurology 102:3341. 10.1212/WNL.0000000000205072

[10] Vetterick C, Lyons KE, Matthews LG et al (2022) The Hidden Burden of Disease and Treatment Experiences of Patients with Essential Tremor: A Retrospective Claims Data Analysis. Adv Ther 39:5546–5567. 10.1007/s12325-022-02318-8 36239902 10.1007/s12325-022-02318-8 PMC9618517

[11] Pouratian N, Baltuch G, Elias WJ, Gross R (2020) American Society for Stereotactic and Functional Neurosurgery Position Statement on Magnetic Resonance-Guided Focused Ultrasound for the Management of Essential Tremor. Neurosurgery 87:E126–E129. 10.1093/neuros/nyz510 31832649 10.1093/neuros/nyz510

[12] Chua MMJ, LeBlang S, Powlovich L et al (2024) Brain targeting for focused ultrasound essential tremor ablation: proceedings from the 2023 focused ultrasound foundation workshop. Neurosurg Focus 57:E3. 10.3171/2024.6.FOCUS2494 10.3171/2024.6.FOCUS2494 39217630

[13] Jung I-H, Chang KW, Park SH et al (2022) Complications after deep brain stimulation: a 21-year experience in 426 patients. Front Aging Neurosci 14. 10.3389/fnagi.2022.819730 10.3389/fnagi.2022.819730 PMC9022472 35462695

[14] Magnetic Resonance-Guided Focused Ultrasound Neurosurgery for Essential Tremor A Health Technology Assessment - PMC. https://pmc.ncbi.nlm.nih.gov/articles/PMC5963668/utm. Accessed 27 Jan 2026 PMC5963668 29805721

[15] Britton TC, Thompson PD, Day BL et al (1993) Modulation of postural tremors at the wrist by supramaximal electrical median nerve shocks in essential tremor, Parkinson’s disease and normal subjects mimicking tremor. J Neurol Neurosurg Psychiatry 56:1085–1089. 10.1136/jnnp.56.10.1085 8410007 10.1136/jnnp.56.10.1085 PMC1015237

[16] Lin PT, Ross EK, Chidester P et al (2018) Noninvasive neuromodulation in essential tremor demonstrates relief in a sham-controlled pilot trial. Mov Disord 33:1182–1183. 10.1002/mds.27350 29663525 10.1002/mds.27350 PMC6174932

[17] Lora-Millan JS, Delgado-Oleas G, Benito-León J, Rocon E (2021) A review on wearable technologies for tremor suppression. Front Neurol 12. 10.3389/fneur.2021.700600 10.3389/fneur.2021.700600 PMC8380769 34434161

[18] Ondo WG, Lv W, Zhu X et al (2025) Transcutaneous Peripheral Nerve Stimulation for Essential Tremor: A Randomized Clinical Trial. JAMA Neurol 82:1235. 10.1001/jamaneurol.2025.3905 41114984 10.1001/jamaneurol.2025.3905 PMC12687095

[19] Dai D, Fernandes J, Kim H, Coetzer H (2023) Comparative Effectiveness of Transcutaneous Afferent Patterned Stimulation Therapy for Essential Tremor: A Randomized Pragmatic Clinical Trial. TOHM 13:38. 10.5334/tohm.798 37869579 10.5334/tohm.798 PMC10588491

[20] Dewey R, Isaacson S, Dewey R et al (2025) A Pilot Study of AI-Controlled Transcutaneous Peripheral Nerve Stimulation for Essential Tremor. Tremor Other Hyperkinet Mov (N Y) 15:10. 10.5334/tohm.991 40125447 10.5334/tohm.991 PMC11927668

[21] Isaacson SH, Peckham E, Tse W et al (2020) Prospective home-use study on non-invasive neuromodulation therapy for essential tremor. Tremor Other Hyperkinetic Movements 10. 10.5334/tohm.59 10.5334/tohm.59 PMC7427656 32864188

[22] Pahwa R, Dhall R, Ostrem J et al (2019) An Acute Randomized Controlled Trial of Noninvasive Peripheral Nerve Stimulation in Essential Tremor. Neuromodulation 22:537–545. 10.1111/ner.12930 30701655 10.1111/ner.12930 PMC6766922

[23] Samiee R, Jameie M, Rahmati M et al (2025) Short-term efficacy of peripheral nerve stimulation for essential tremor in a randomized double-blind controlled trial. Sci Rep 15:28713. 10.1038/s41598-025-13487-1 40770217 10.1038/s41598-025-13487-1 PMC12328561

[24] Page MJ, McKenzie JE, Bossuyt PM et al (2021) The PRISMA 2020 statement: an updated guideline for reporting systematic reviews. BMJ 372:n71. 10.1136/bmj.n71 33782057 10.1136/bmj.n71 PMC8005924

[25] Higgins JPT, Thomas J, Chandler J et al (2019) Cochrane handbook for systematic reviews of interventions. 10.1002/9781119536604 10.1002/14651858.ED000142 PMC10284251 31643080

[26] Amir-Behghadami M, Janati A (2020) Population, Intervention, Comparison, Outcomes and Study (PICOS) design as a framework to formulate eligibility criteria in systematic reviews. Emerg Med J 37:387. 10.1136/emermed-2020-209567 32253195 10.1136/emermed-2020-209567

[27] Ouzzani M, Hammady H, Fedorowicz Z, Elmagarmid A (2016) Rayyan—a web and mobile app for systematic reviews. Syst Reviews 5:210. 10.1186/s13643-016-0384-4 10.1186/s13643-016-0384-4 PMC5139140 27919275

[28] Higgins JPT, Savović J, Page MJ et al (2019) Assessing risk of bias in a randomized trial. Cochrane Handbook for systematic reviews of interventions. Wiley, Ltd, pp 205–228

[29] McGuinness LA, Higgins JPT (2021) Risk-of-bias VISualization (robvis): An R package and Shiny web app for visualizing risk-of-bias assessments. Res Synthesis Methods 12:55–61. 10.1002/jrsm.1411 10.1002/jrsm.1411 32336025

[30] Study Quality Assessment Tools | NHLBI, NIH. https://www.nhlbi.nih.gov/health-topics/study-quality-assessment-tools. Accessed 10 Mar 2025

[31] Schünemann HJ, Higgins JPT, Vist GE et al (2019) Completing ‘Summary of findings’ tables and grading the certainty of the evidence. Cochrane Handbook for systematic reviews of interventions. Wiley, Ltd, pp 375–402

[32] Murad MH, Mustafa RA, Schünemann HJ et al (2017) Rating the certainty in evidence in the absence of a single estimate of effect. Evid Based Med 22:85–87. 10.1136/ebmed-2017-110668 28320705 10.1136/ebmed-2017-110668 PMC5502230

[33] Elble RJ (2016) The essential tremor rating assessment scale. J Neurol Neuromedicine 1(4):34–3828405636

[34] Higgins JPT, Li T, Deeks JJ (2019) Choosing effect measures and computing estimates of effect. Cochrane Handbook for systematic reviews of interventions. Wiley, Ltd, pp 143–176

[35] AydinO, Yassikaya MY (2021) Validity and reliability analysis of the plotdigitizer software program for data extraction from single-case graphs. Perspect Behav Sci 45:239–257. 10.1007/s40614-021-00284-0 35342869 10.1007/s40614-021-00284-0 PMC8894524

[36] Egger M, Smith GD, Schneider M, Minder C (1997) Bias in meta-analysis detected by a simple, graphical test. BMJ 315:629–634. 10.1136/bmj.315.7109.629 9310563 10.1136/bmj.315.7109.629 PMC2127453

[37] Pascual-Valdunciel A, Gonzalez-Sanchez M, Muceli S et al (2021) Intramuscular Stimulation of Muscle Afferents Attains Prolonged Tremor Reduction in Essential Tremor Patients. IEEE Trans Biomed Eng 68:1768–1776. 10.1109/TBME.2020.3015572 32813648 10.1109/TBME.2020.3015572

[38] Yu JY, Rajagopal A, Syrkin-Nikolau J et al (2020) Transcutaneous Afferent Patterned Stimulation Therapy Reduces Hand Tremor for One Hour in Essential Tremor Patients. Front Neurosci 14:530300. 10.3389/fnins.2020.530300 33281539 10.3389/fnins.2020.530300 PMC7689107

[39] Kim J, Wichmann T, Inan OT, DeWeerth SP (2022) Analyzing the effects of parameters for tremor modulation via phase-locked electrical stimulation on a peripheral nerve. JPM 12:76. 10.3390/jpm12010076 10.3390/jpm12010076 PMC8779889 35055390

[40] Munhoz RP, Hanajima R, Ashby P, Lang AE (2003) Acute effect of transcutaneous electrical nerve stimulation on tremor. Mov Disord 18:191–194. 10.1002/mds.10311 12539214 10.1002/mds.10311

[41] Luu CP, Ranum J, Youn Y et al (2025) Wearable peripheral nerve stimulator reduces essential tremor symptoms through targeted brain modulation. Brain Stimul 18:1162–1173. 10.1016/j.brs.2025.06.004 40484122 10.1016/j.brs.2025.06.004

[42] Barath AS, Rusheen AE, Min H-K et al (2020) Brain Metabolic Changes with Longitudinal Transcutaneous Afferent Patterned Stimulation in Essential Tremor Subjects. Tremor Other Hyperkinetic Movements 10:52. 10.5334/tohm.565 33362946 10.5334/tohm.565 PMC7747758

[43] Kachhadia MP, Sibhai I, Vaghela R et al. Open-loop and closed-loop neuromodulation across neurological disorders toward personalized brain stimulation. Narrative Rev Cureus 17:e98624. 10.7759/cureus.98624 10.7759/cureus.98624 PMC12771547 41503301

[44] Hollý P, Hubená T, Čihák M et al (2024) Estimating Disability in Patients with Essential Tremor: Comparison of Tremor Rating Scale, Spiral Drawing, and Accelerometric Tremor Power. Mov Disord Clin Pract 11:1582–1586. 10.1002/mdc3.14160 38989643 10.1002/mdc3.14160 PMC11647970

[45] Nicoletti V, Cecchi P, Pesaresi I et al (2020) Cerebello-thalamo-cortical network is intrinsically altered in essential tremor: evidence from a resting state functional MRI study. Sci Rep 10:16661. 10.1038/s41598-020-73714-9 33028912 10.1038/s41598-020-73714-9 PMC7541442

[46] Luft AR, Manto M-U, Ben Taib NO (2005) Modulation of motor cortex excitability by sustained peripheral stimulation: the interaction between the motor cortex and the cerebellum. Cerebellum 4:90–96. 10.1080/14734220410019084 16035190 10.1080/14734220410019084

[47] Gironell A, Marín-Lahoz J, Póveda S (2024) Essential tremor: Update of therapeutic strategies. Med Clínica (English Edition) 162:599–605. 10.1016/j.medcle.2023.12.015 10.1016/j.medcli.2023.12.013 38553256

[48] Ferreira JJ, Mestre TA, Lyons KE et al (2019) MDS evidence-based review of treatments for essential tremor. Mov Disord 34:950–958. 10.1002/mds.27700 31046186 10.1002/mds.27700

[49] Antonazzo IC, Rozza D, Conti S et al (2024) Treatment patterns in essential tremor: Real-world evidence from a United Kingdom and France primary care database. Euro J Neurol 31:e16064. 10.1111/ene.16064 10.1111/ene.16064 PMC11235796 37738526

[50] Dash D, Bruno V, Schwingenschuh P et al (2026) Update on medical treatments for essential tremor: an international parkinson and movement disorder society evidence-based medicine review. Movement Disorders mds.70184 10.1002/mds.70184 10.1002/mds.70184 PMC13067321 41556478

[51] Brillman S, Colletta K, Borucki S et al (2022) Real-World Evidence of Transcutaneous Afferent Patterned Stimulation for Essential Tremor. Tremor Other Hyperkinetic Movements 12:27. 10.5334/tohm.715 36119968 10.5334/tohm.715 PMC9442494

[52] Lu C, Khosla D, Kent A et al (2023) Transcutaneous Afferent Patterned Stimulation for Essential Tremor: Real-World Evidence with Long Term Follow-Up. Tremor Other Hyperkinetic Movements 13:29. 10.5334/tohm.775 37663529 10.5334/tohm.775 PMC10473165

[53] Zhang J, Yan R, Cui Y et al (2024) Treatment for essential tremor: a systematic review and Bayesian Model-based Network Meta-analysis of RCTs. eClinicalMedicine 77:102889. 10.1016/j.eclinm.2024.102889 39498461 10.1016/j.eclinm.2024.102889 PMC11533039

[54] Agrawal M, Garg K, Samala R et al (2021) Outcome and Complications of MR Guided Focused Ultrasound for Essential Tremor: A Systematic Review and Meta-Analysis. Front Neurol 12:654711. 10.3389/fneur.2021.654711 34025558 10.3389/fneur.2021.654711 PMC8137896

[55] Deshpande N, Hadi M, Ali R (2024) Healthcare disparities in deep brain stimulation access and utilization: a systematic review. J Neurosurg 140:1137–1147. 10.3171/2023.7.JNS23277 38240596 10.3171/2023.7.JNS23277

[56] Venkatraman V, Futch BG, Bode Padron KJ et al (2024) Disparities in the treatment of movement disorders using deep brain stimulation. J Neurosurg 141:241–251. 10.3171/2023.11.JNS23882 38306639 10.3171/2023.11.JNS23882 PMC10898494

[57] Coltman SK, Hu X (2025) Minimizing sensory habituation in nerve stimulation through strategic temporal stimulation patterns. IEEE J Biomedical Health Inf 1–10. 10.1109/JBHI.2025.3597855 10.1109/JBHI.2025.3597855 40794499

[58] Cosgrove GR, Lipsman N, Lozano AM et al (2023) Magnetic resonance imaging–guided focused ultrasound thalamotomy for essential tremor: 5-year follow-up results. J Neurosurg 138:1028–1033. 10.3171/2022.6.JNS212483 35932269 10.3171/2022.6.JNS212483 PMC10193464

