# Supplementary material for: Peripheral nerve stimulation for essential tremor: a GRADE-assessed systematic review and meta-analysis
Source: Neurosurg Rev. 2026 Jul 21;49(1):486. doi: 10.1007/s10143-026-04394-8 (PMC13385257; doi:10.1007/s10143-026-04394-8)
Supplement: Supplementary file 1 — Supplementary Material 1 (DOCX 1.58 MB) [file 10143_2026_4394_MOESM1_ESM.docx]

**Contents**

[Table 1 Search Strategy 2](#_Toc221729713)

# Table 1 Search Strategy

| **Data base** | **Search strategy** | **Number of studies** |
| --- | --- | --- |
| PubMed | 1 = (Transcutaneous afferent patterned stimulation) OR (Transcutaneous peripheral nerve stimulation) OR (Peripheral Nerve Stimulation) OR (TAPS) OR (AI-TAPS) OR (PNS) OR (TPNS) OR (intramuscular electrical stimulation) AND (Essential tremors) OR (Action tremor) OR (Postural tremor)  2 = ("Essential Tremor"[Mesh]) AND "Transcutaneous Electric Nerve Stimulation"[Mesh] | 72 |
| Scopus | ( "Transcutaneous afferent patterned stimulation" OR "Transcutaneous peripheral nerve stimulation" OR "Transcutaneous Electric Nerve Stimulation" OR "Peripheral Nerve Stimulation" OR "intramuscular electrical stimulation" OR "TAPS" OR "PNS" OR "TPNS" ) AND ( "Essential tremors" OR "Essential tremor" OR "Action tremor" OR "Postural tremor" ) | 66 |
| WOS |  | 56 |
| Cochrane |  | 31 |
